# Cardiothoracic Transplant Surgery and Enhanced Recovery: Recent Advances and Perspectives

**DOI:** 10.3390/jcm15031179

**Published:** 2026-02-03

**Authors:** Riya Aggarwal, Jeremiah Hutson, David Zapata, Howard Massey, Bradley Taylor, Bartley Griffith, Justin Robinson

**Affiliations:** 1 College of Human Medicine, Michigan State University, Grand Rapids, MI 49503, USA; aggarw51@msu.edu (R.A.); hutsonj2@msu.edu (J.H.); 2 Department of Surgery, University of Maryland School of Medicine, Baltimore, MD 21201, USA; dzapata@som.umaryland.edu (D.Z.); hmassey@som.umaryland.edu (H.M.); taylorb2@mac.com (B.T.); bgriffith@som.umaryland.edu (B.G.)

**Keywords:** enhanced recovery after surgery, heart transplant, lung transplant, cardiothoracic transplantation, cardiothoracic surgery

## Abstract

**Introduction**: Cardiothoracic transplant surgery represents a critical intervention for patients with end-stage heart and/or lung failure. While advancements in surgical techniques and perioperative management have enhanced survival rates, these procedures remain associated with significant morbidity, extended hospitalizations, and complex recovery trajectories. **Background/Objectives**: Enhanced Recovery After Surgery (ERAS) protocols, originally developed for colorectal surgery, have shown promise in optimizing perioperative care across various surgical disciplines. However, their application in cardiac and thoracic transplantation is still emerging. This article evaluates recent advancements in ERAS protocols tailored to cardiac and thoracic transplant patients, focusing on preoperative, intraoperative, and postoperative interventions. **Results**: Evidence highlights the potential of ERAS to reduce complications, shorten hospital stays, and improve long-term outcomes. Key strategies include preoperative optimization through nutritional and psychosocial prehabilitation, intraoperative adoption of minimally invasive techniques and refined anesthesia practices, and postoperative protocols emphasizing opioid-sparing pain management, early mobilization, and nutritional recovery. **Conclusions**: This review identifies gaps in current research and offers recommendations for the broader implementation and standardization of ERAS protocols in cardiothoracic surgery, with emphasis on cardiothoracic transplantation, aiming to improve outcomes for this high-risk population.

## 1. Introduction

Heart and lung transplantation are lifesaving interventions for patients with end-stage organ failure. These operations are inherently risky, and are associated with considerable morbidity, prolonged hospital stays, costs, and extensive recovery periods. Mortality rates regarding transplantation are high; In 2020, estimated 1 and 5-year survival was 85% and 59% following lung transplantation [1] and 85% and 73% for heart transplant respectively [2]. Importantly, some opportunities to decrease mortality and rejection rates can involve patient protocols implemented before, during, and after surgery.

The importance of multifactorial operative challenges has led to the growing adoption of ERAS protocols: a multidisciplinary approach designed to optimize perioperative care and accelerate recovery. These strategies were initially developed for colorectal surgery [3] but have since been adapted across a wide range of surgical specialties, including cardiothoracic surgery [4]. These evidence-based pathways integrate strategies such as preoperative patient education, nutritional optimization, minimally invasive surgical techniques, multimodal analgesia, and early mobilization. By minimizing the physiological stress of surgery and standardizing care delivery, ERAS protocols aim to enhance clinical outcomes, reduce complications, decrease hospital costs [5,6], and improve patient satisfaction.

Despite the established success of ERAS in general surgery, its implementation in cardiac and thoracic transplantation has been limited. The unique challenges of cardiac and thoracic transplant surgery—including the complexity of the procedure, the vulnerability of immunosuppressed patients, and the risk of organ rejection—necessitate tailored ERAS pathways. A successful implementation of ERAS protocols within cardiac and thoracic transplant surgery is possible, and a broad-based, multidisciplinary approach is imperative for success [7]. By synthesizing the latest evidence, this review aims to identify best practices, highlight current gaps in knowledge, and propose future directions for research and clinical implementation. Due to the relative deficiency of ERAS cardiac and lung transplantation literature, we supplemented limited existing literature with well-studied cardiothoracic techniques. Through this effort, we hope to contribute to the evolving paradigm of perioperative care in cardiac and thoracic transplant surgery, ultimately improving outcomes for this high-risk patient population.

## 2. Materials and Methods

### 2.1. Study Design and Search Strategy

**Figure 1:**
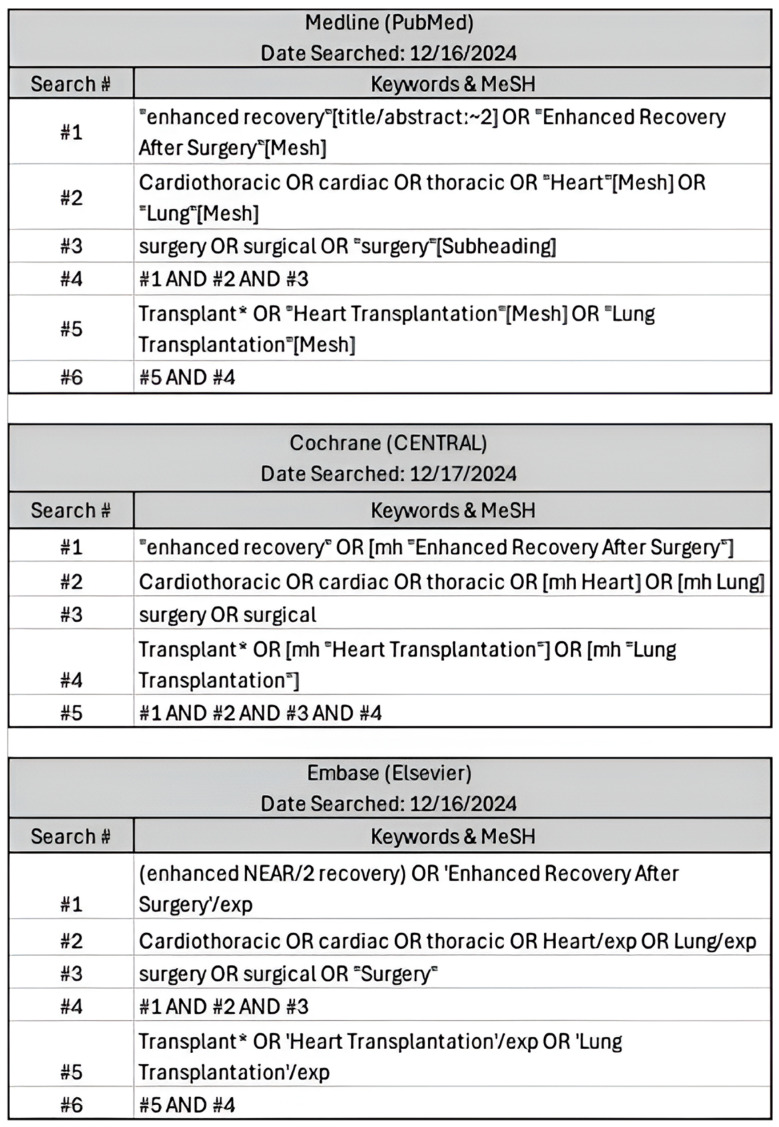
Comprehensive search inputs for PubMed, Cochrane Central, Elsevier with keywords used and respective search engine terms. Search criteria were defined and translated into steps ranging from 5-6 search items to ensure similar parameters were searched between engines.

This systematic review and meta-analysis was conducted in accordance with the Preferred Reporting Items for Systematic Reviews and Meta-Analyses (PRISMA) guidelines [8] (Supplementary Materials S1). A comprehensive search was conducted in Medline (PubMed), Cochrane Central, and Embase (Elsevier) in December 2024 to identify relevant studies published. The search strategy incorporated a combination of Medical Subject Headings (MeSH) terms and free-text keywords. These included terms such as “cardiothoracic,” “heart transplant,” “lung transplant,” “enhanced recovery,” “Enhanced Recovery After Surgery,” and “surgery” and relevant synonyms (Figure 1). Boolean operators (AND, OR) were applied to refine search results.

### 2.2. Eligibility Criteria

(a) Study Design:
  - Original studies
  - Observational studies (e.g., cohort, case-control, and cross-sectional studies)
  - Randomized Controlled Trials (RCTs)
  - Non-Randomized Controlled Trials (Non-RCTs)
  - Retroactive/Prospective cohort studies
  - Literature reviews, summaries, official recommendations, and commentaries
(b) Outcomes:
  - Primary—preoperative, intraoperative, postoperative cardiothoracic transplantation ERAS techniques
  - Secondary—cardiothoracic ERAS techniques
(c) Language:
  - Only studies published in English were included
(d) Population:
  - Studies involving adult patients (aged 18 years and above) who have undergone cardiothoracic surgery.
(e) Exclusion Criteria:
  - Studies that do not focus on enhanced recovery after surgery (ERAS)
  - Studies involving pediatric populations
  - Studies involving non-human specimens
  - Studies on patients undergoing non-thoracic surgeries
  - Studies without clear methodological details.

### 2.3. Data Extraction and Inspection

**Figure 2:**
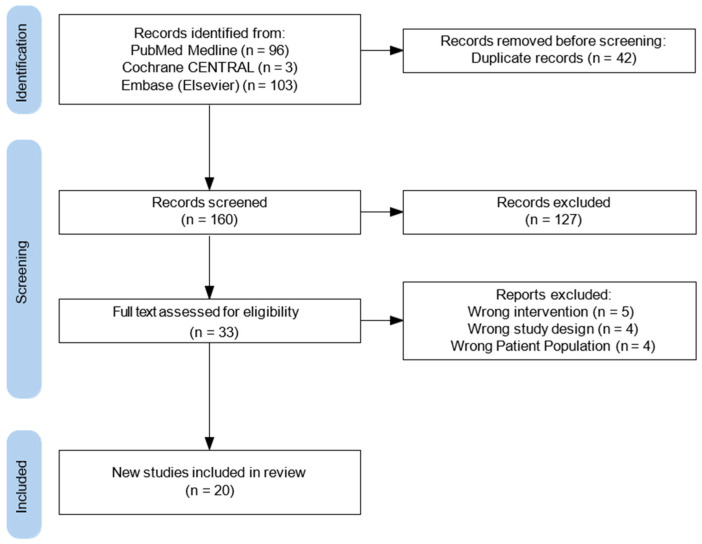
Flow diagram of study selection process.

All search results were imported into an online systematic review management platform, which facilitated study screening, duplication, and data organization. Two independent reviewers (RA, JH) conducted title and abstract screening based on predefined inclusion and exclusion criteria. Full-text screening was performed for articles deemed potentially eligible. Discrepancies between reviewers during either phase were resolved through discussion or consultation with a third reviewer (JR). A flow diagram was generated to visualize the study selection process (Figure 2). Two reviewers (RA, JH) independently performed data extraction, and any inconsistencies were resolved by consensus decision. Findings were organized into preoperative, intraoperative, and postoperative components of enhanced recovery protocols. Ultimately twenty studies were included for data extraction, with a mixture of cardiothoracic transplantation and established cardiothoracic literature. This literature includes studies from 2018 to 2024.

### 2.4. Risk of Bias/Quality Assessment

The methodological quality of the included studies was assessed using Covidence’s default quality assessment Cochrane Risk of Bias tool. Quality assessment was performed by Covidence with predefined scoring criteria. Overall, each study was evaluated for risk of bias, validity, and relevance to the review objectives by the previous tools as well as within review of the three reviewers.

## 3. Results

### 3.1. Study Selection

During the initial database search, which utilized a predefined set of relevant keywords and phrases, a total of 202 studies were identified. After duplicates were removed—accounting for 42 entries—the dataset was refined to ensure accuracy and relevance for further analysis. Following this step, 160 articles remained for additional screening, as outlined in Figure 2. During the screening process, 127 studies were excluded for several reasons: they were not full-text original studies, they consisted of review articles, letters, editorials, or systematic reviews, or they focused on unrelated topics such as non-cardiothoracic transplant surgeries (i.e. liver, colorectal, or renal transplants) or they were not pertinent to ERAS. Ultimately, 33 original studies met the eligibility criteria after the full screening process (Figure 2). Among the eligible studies, five did not focus on the correct intervention, four utilized the wrong study design, and four focused on the wrong patient population. As a result, 20 studies met all inclusion criteria by employing validated methodologies to evaluate enhanced recovery protocols in cardiothoracic transplant surgery (Table 1) (Appendix A).

### 3.2. Preoperative Strategies

Preoperative optimization is a cornerstone of enhanced recovery pathways, aiming to prepare patients for the challenges of cardiac and/or thoracic transplantation. Current evidence highlights the importance of prehabilitation strategies, including nutritional supplementation, smoking cessation, and physical conditioning, particularly in lung transplantation. For heart transplantation, there is a notable gap in research specifically addressing preoperative ERAS protocols. However, we propose that findings from lung transplantation and cardiac surgery suggest that similar strategies could be extrapolated to heart transplant patients.

#### 3.2.1. Team Selection and Preadmission

The multidisciplinary team typically includes cardiac surgeons, cardiologists, anesthesiologists, intensivists, perfusionists, cardiac rehabilitation specialists, nurses, allied health professionals, pharmacists, and dietitians [4]. The involvement of these specialists, alongside a dedicated coordinator, ensures the full buy-in of pertinent stakeholders for the success and implementation of ERAS programs [27] and that all aspects of the patient’s health are addressed, from optimizing cardiac and pulmonary function to providing tailored nutritional and psychological support.

Preoperative counseling is instrumental in providing patients with verbal explanations, informational packets, and multimedia resources. These detail the procedure and include cognitive strategies that enhance pain management, minimize nausea and anxiety post-surgery, improve outcomes following general anesthesia, and foster a sense of preparedness [21,25,26]. Patients and caregivers should receive detailed guidance on perioperative expectations, rehabilitation goals, and the importance of adherence to prescribed regimens.

Additionally, recent research in lung transplantation highlights the significance of preoperative anesthesia consultations in outlining the stages of transplantation and introducing complementary techniques to support patients during potentially painful aspects of the procedure. This holistic approach empowers patients to actively participate in their own care by equipping them with personalized tools such as self-hypnosis, relaxation techniques, sophrology, or transcutaneous electrical nerve stimulation to manage their experience effectively [28].

#### 3.2.2. Preoperative Screening and Optimization

Preoperative screening is a critical component of enhanced recovery protocols, ensuring that patients are optimally prepared for the demands of cardiac and/or thoracic transplantation. Screening for frailty using tools such as the Lung Transplant Frailty Scale in preparation for prehabilitation allows clinicians to identify vulnerable patients who may require additional interventions to improve strength and postoperative outcomes [9,12]. Preoperative screening for malnutrition is crucial as it helps identify patients who are at risk and could benefit from targeted nutritional interventions [9]. Tools such as the Nutritional Risk Score, the Malnutrition Universal Screening Tool and the Subjective Global Assessment have been shown to be useful [29]. These screening tools, assessing BMI, recent weight loss, and overall nutritional status, are essential to targeting nutritional prehabilitation, discussed in the next section.

Preoperative anemia is linked to increased surgery risks, mortality, morbidity, and reduced long-term survival [25]. Anemia should be screened and treated at least 4 weeks prior to surgery as it has been shown to have the potential to reduce the risks associated with surgery and improve clinical outcomes including postoperative morbidity, mortality as well as prolonging length of hospital stay [30]. Additionally, correcting anemia preoperatively reduces the risk of complications associated with anemia itself or the need for blood transfusions. Correction for anemia has been shown to be effective in the immediate perioperative period [31].

Finally, for patients undergoing thoracic transplant surgery, comprehensive screening of comorbidities, including renal insufficiency, diabetes, and cardiovascular disease, ensures these conditions are fully evaluated and optimized prior to surgery [16,20]. For diabetic patients, monitoring hemoglobin A1c levels and achieving glycemic control with an A1c level below 6.5–7% has been associated with significant decreases in deep sternal wound infections, ischemic events, and additional adverse events [7,32].

#### 3.2.3. Prehabilitation, Nutrition, and Supplementation

Prehabilitation is a critical component of the enhanced recovery pathway for both heart and lung transplantation patients. This encompasses the importance of psychosocial support, exercise, smoking cessation, improved respiratory function, nutrition optimization, and more. Tobacco use and excessive alcohol consumption are well-established significant risk factors for postoperative complications, including respiratory issues, delayed wound healing, bleeding, metabolic imbalances, and infections [33]. Interventions targeting these behaviors before surgery offer a valuable opportunity to improve outcomes. Evidence suggests that quitting smoking and abstaining from alcohol for at least one month prior to surgery can lead to better postoperative results [7,25]. Although limited research exists specific to cardiothoracic surgery, the minimal risk of these interventions makes them essential.

Following the screening processes outlined in the previous section, a multimodal prehabilitation program aims to identify physical, nutritional, and psychological factors that are reversible. Prehabilitation exercise programs should be designed using the “FITT” principle, which incorporates frequency, intensity, time (duration), and type of exercise as key components. Incorporating technology, such as accelerometers, to objectively monitor exercise progress can provide valuable feedback and motivation [28]. Aerobic and resistance training programs tailored to the patient’s functional capacity have been demonstrated to improve postoperative recovery, reduce complications, and shorten hospital stays [25]. Additionally, inspiratory muscle training has been shown to improve respiratory muscle strength and endurance, thus reducing postoperative cardiopulmonary complications [4,34]. Psychosocial prehabilitation is equally important to health optimization pre-transplant. Evidence suggests the importance of creating a patient-centered, tailored prehabilitation plan through supportive behavioral therapies and improved mental well-being. It is proposed that this can be through the creation of a calm and benevolent atmosphere, where the multi-disciplinary team allows the patient to choose the music in the operating room and catered every detail to the individual patient’s objective disease and subjective preferences [12].

Finally, targeted nutritional interventions, such as the administration of oral supplements to ensure patients consume 1.2–2.0 g of protein per kilogram of body weight daily, can mitigate the metabolic stress of surgery and promote healing [9]. Recent evidence supports allowing patients to consume 200 mL of a clear carbohydrate drink (24 g of glucose) two hours before surgery, challenging the traditional fasting guidelines established by the American Society of Anesthesiologists over two decades ago [21]. This carbohydrate-loading strategy helps prevent protein catabolism and has gained popularity for its metabolic benefits [14]. In thoracic surgery, fluid management has traditionally followed a volume-restrictive approach, with intraoperative and postoperative maintenance fluids limited to 1–2 mL/kg/h and a perioperative fluid balance of less than 1500 mL (or 20 mL/kg/24 h) [25]. This strategy aims to minimize pulmonary capillary hydrostatic pressure. However, overly restrictive fluid management can lead to hypovolemia, impaired tissue perfusion, organ dysfunction, and acute kidney injury [35]. Once again, though there is established evidence in lung transplant surgery and general cardiac surgery, research specific to heart transplant is minimal and a necessary gap to be filled.

#### 3.2.4. Management of Primary Graft Dysfunction and Prolonged Ischemia Time

Primary graft dysfunction (PGD) and prolonged ischemia time remain significant challenges in cardiac and thoracic transplantation, contributing to early morbidity and mortality in recipients. PGD, a severe form of acute lung or heart injury occurring within the first 72 h post-transplant, is associated with poor graft function and systemic complications. Prolonged ischemia time, defined as the duration between organ procurement and reperfusion, is a major risk factor for PGD and directly impacts graft viability and recipient outcomes. These issues highlight the need for evidence-based strategies to mitigate ischemic injury and optimize graft performance before, during, and after transplantation.

In 2019, the PREDICTA score was introduced as a novel tool for predicting PGD risk in first-time heart transplant recipients [36]. This scoring system considers recipient factors (preoperative mechanical circulatory support and diabetes), procedural factors (cardiopulmonary bypass and implant times), and donor age, incorporating modern practices like donation after circulatory death and ex vivo normothermic perfusion [13]. There is also some evidence to suggest the management of PGD through prompt Extracorporeal Membrane Oxygenation (ECMO) implementation. Additionally, recent advancements in controlled hypothermic storage in the range of 4 °C to 10 °C show promise in extending preservation times, minimizing ischemic time to maintain graft viability, especially in lung and heart transplantation patients [4,37].

#### 3.2.5. Pain Management, Antibiotic Prophylaxis, and Surgical Preparation

Lung transplant and cardiac surgery ERAS research has demonstrated the importance of adequately controlling pain, minimizing infection, and optimizing surgical preparation. Once again, though limited evidence exists for cardiac transplantation specifically, we believe that these interventions can be extrapolated with minimal to no harm to patients undergoing heart transplant. Opioid-sparing pain management through the use of multimodal analgesia techniques is a core foundation of enhanced recovery practices across all surgical subspecialty populations [4]. Studies demonstrate the most benefit following an ERAS protocol that includes preemptive analgesia with 300–600 mg gabapentin and 650–1000 mg acetaminophen orally pre-transplant 30 min before anesthesia induction or 2 h prior to surgery [21,24,26]. This procedure has demonstrated decreased median pain scores and minimize postoperative opioid usage.

Preoperative antibiotic prophylaxis and optimal skin preparation are critical components of ERAS protocols to minimize surgical site infections in cardiac and thoracic transplantation. Administering intravenous antibiotic prophylaxis, intranasal therapies to eradicate staphylococcal colonization, and weight-based cephalosporins such as 2 g of cefazolin or 1 g of vancomycin, within 30–60 min before the skin incision and continued for 48 h after completion of cardiothoracic surgery is a practice that has been shown to significantly reduce postoperative infection risk [7,25,26,38]. In cases requiring hair removal, hair clipping is preferred over shaving to minimize microtrauma and potential contamination. Additionally, chlorhexidine–alcohol has been demonstrated to be more effective than povidone-iodine for skin preparation, further enhancing infection control [10,21,27]. By incorporating these evidence-based practices, ERAS protocols aim to improve surgical outcomes, reduce complications, and promote faster recovery in transplant patients.

### 3.3. Intraoperative Practices

The intraoperative phase of cardiothoracic transplantation plays a pivotal role in determining surgical outcomes and long-term graft viability. This period demands meticulous coordination of surgical techniques, anesthesia, and perfusion strategies to address the complexities of transplantation. Advances in intraoperative practices, including refined surgical methods, enhanced multimodal pain management strategies, and optimized extracorporeal life support (ECLS) protocols, have significantly improved patient outcomes. Additionally, the management of bleeding and hemodynamics, coupled with tailored anesthesia techniques, has become essential in reducing perioperative complications and enhancing recovery. However, there remains a notable gap in evidence and research specific to heart transplantation, highlighting the need for further studies to guide and optimize intraoperative management in this population. This section reviews the latest evidence and practices in intraoperative management, providing insights into their role in improving the success of cardiac and thoracic transplantation.

#### 3.3.1. Surgical Techniques

In contrast with the conventional transverse thoracosternotomy, minimally invasive lung transplantation (MILT) both reduces exposure of the patient to the operating environment and has goals in decreasing recovery time of the patient. MILT offers multiple benefits, including reduced recovery times, shorter ICU stays, and decreased opioid requirements at discharge. Importantly, studies indicate that MILT patients achieve earlier improvements in respiratory function, as measured by forced expiratory volume in one second [39]. These findings underscore MILT with full implementation of an ERAS protocol as a safe and effective alternative to conventional approaches for lung transplantation, offering earlier improvement in patient respiratory function and outcomes [16,23].

#### 3.3.2. Intraoperative Multimodal Pain Management

Opioid analgesics have many adverse effects detrimental to post-thoracic transplant recovery, namely sedation, respiratory depression, nausea, and vomiting [15]. Additionally, opioids are associated with increased risk of graft failure and mortality in many transplant populations, including lung transplant recipients [40]. As demonstrated for preoperative ERAS protocols in transplantation, intraoperative opioid-sparing, multimodal pain regimens are paramount to increasing patient outcomes, decreasing length of stay, and ultimately decreasing morbidity and mortality. Studies demonstrate that the usage of 266 mg liposomal bupivacaine intercostal nerve block LB-INB (20 mL) diluted in 20 mL normal saline in the operating room around the fifth intercostal space following chest closure decreased postoperative opioid consumption significantly [15,24]. These findings have been established for lung transplantation recipients but there is no current evidence in the heart transplantation patient population.

#### 3.3.3. ECLS Management

The use of hybrid veno-arterial extracorporeal membrane oxygenation circuits has become increasingly valuable, providing hemodynamic support while facilitating lung-protective ventilation strategies to minimize ventilator-induced lung injury [12,41]. In parallel, electroencephalogram and hematological monitoring combined with cerebral oximetry ensures optimal cerebral perfusion and oxygenation during the procedure, reducing risk of any possible neurological complications [42].

Additionally, the strict post-cardiopulmonary bypass temperature control, maintaining normothermia within the range of 36.5 °C to 37.2 °C and avoiding hyperthermia when rewarming on CPB, is essential for preserving metabolic stability and improving patient outcomes [7,10,21]. Maintaining euvolemia is equally critical, as precise fluid management supports optimal organ perfusion and prevents complications such as pulmonary edema or acute kidney injury [14].

#### 3.3.4. Bleeding and Anesthesia Techniques

Point-of-care coagulation testing establishes a specific algorithm based on rotational thromboelastometry and platelet aggregometry assays to the standard of care. With this implementation, point-of-care coagulation testing not only decreases overall transfusion cost, but also results in less perioperative blood loss and blood product consumption [12].

Additionally, antifibrinolytic use with tranexamic acid and epsilon aminocaproic acid is recommended during on-pump cardiac surgical procedures. These medications have been demonstrated to reduce total blood products transfused, and major hemorrhage or tamponade requiring reoperation [7,43]. Furthermore standardized short-acting anesthetics in cardiac surgery are utilized with the ERAS protocols. These not only allow for quicker extubation of post-op patients but also aid in reducing the many other complications of long-acting anesthetics on patients [21].

### 3.4. Postoperative Care

Postoperative care is a critical phase in ERAS protocols for cardiothoracic transplantation, focusing on optimizing recovery, minimizing complications, and promoting long-term graft function. This phase encompasses a multidisciplinary approach that includes multimodal pain management to reduce opioid reliance, homeostatic maintenance to stabilize vital functions, and tailored nutritional strategies to support healing and immune function. Effective ECLS management and interventions for ischemic reperfusion injury at the postoperative stage play pivotal roles in preserving organ integrity and preventing graft dysfunction. Additionally, early mobilization, physiotherapy, and psychotherapy are integral to enhancing physical and psychological recovery, empowering patients to achieve better outcomes.

#### 3.4.1. Postoperative Multimodal Pain Management

Opioid-sparing multimodal analgesia reduces pain, enhances recovery, and minimizes opioid-related complications such as respiratory depression, nausea, vomiting, and delayed mobilization [44]. In lung transplantation, preemptive analgesia protocols incorporating gabapentin and acetaminophen, combined with postoperative administration of gabapentin, methocarbamol, and potential serratus anterior plane blocks, have demonstrated success in achieving minimal opioid use while maintaining acceptable pain control [7,11,12,15,24]. These techniques have shown potential for adaptation to cardiac transplantation patients, where effective pain management plays a critical role in facilitating early extubation, reducing recovery time, and minimizing complications such as anorexia, malnutrition, and unnecessary suffering [45].

Postoperative opioid-sparing analgesia protocols for cardiac transplantation can include a combination of acetaminophen, gabapentin or pregabalin, methocarbamol, and Lidoderm patches, with opioids reserved for breakthrough pain. The addition of fascial plane blocks, such as the serratus anterior or erector spinae plane blocks, may provide regional analgesia with promising results, even in patients with coagulation disorders [11,46,47]. Studies highlight the potential of these techniques in reducing opioid requirements and enhancing patient comfort [19,24]. Emerging evidence also supports the inclusion of intravenous acetaminophen (1 g every 8 h), tramadol, dexmedetomidine, and regional analgesia techniques based on individual patient needs and formulary availability postoperatively. There has also been a demonstrated applicability of neuraxial, para-neuraxial, and fascial plane blocks—well-studied in other cardiac surgeries—to heart transplant patients [11].

#### 3.4.2. Homeostatic Maintenance

Body temperature abnormalities are among the most frequently observed symptoms in critically ill patients in the ICU and are strongly associated with increased mortality risk [7,48]. Thus, meticulous temperature management is essential to optimize outcomes and prevent complications. Evidence from cardiac surgery demonstrates that strategies such as forced-air warming blankets, maintaining elevated ambient room temperatures, and pre-warming irrigation and intravenous fluids significantly reduce the incidence of hypothermia and its associated risks. These measures also contribute to enhanced recovery by reducing the risk of infection, coagulopathy, and hemodynamic instability—factors critical to the success of ERAS pathways [7,21].

#### 3.4.3. Nutrition and Postoperative Nausea and Vomiting Management

Re-establishment of oral feeding as early as possible has become a pillar of ERAS protocols, particularly in the context of organ transplantation [49]. Though research is still limited, this provides promise for future application specific to thoracic transplant procedures. Evidence underscores that initiating nutrition promptly postoperatively significantly reduces infectious complications, decreases ICU and hospital stay durations, and lowers overall costs and mortality rates [50].

In lung transplantation, protocols have evolved to minimize the reliance on intravenous fluids, transitioning patients from a liquid diet over two weeks to a soft diet for one week, and then gradually advancing to a full diet, including regular foods, by the fifth postoperative week [9,14,16]. Other methodologies have emphasized early enteral or central parenteral nutrition within the first 48 h following transplantation. These protocols recommend chewing gum four hours after tracheal extubation, proposed to stimulate digestive activity [51]. These strategies, combined with targeted energy and protein goals (e.g., 25 kcal and 1 g of protein per kg of ideal body weight daily by day seven post-procedure), have demonstrated benefits in mitigating weight loss and preserving muscle mass in transplant recipients [22,27]. Non-pharmacological approaches to managing postoperative nausea and vomiting further contribute to improved recovery. Techniques such as controlled breathing exercises, aromatherapy, and acupressure have been shown to alleviate symptoms effectively without the side effects of pharmacological agents [10,25].

#### 3.4.4. Ischemic Reperfusion Injury Management

Ischemic reperfusion injury (IRI) is a pathological process that results in significant tissue damage due to the restoration of blood flow after ischemia; compromising the viability and functionality of transplanted organs. Severe IRI, characterized by diffuse alveolar infiltrates, worsening hypoxemia, and decreased lung compliance within 72 h post-lung transplantation, has shown significant improvement with the administration of exogenous surfactant. In clinical practice, one vial of surfactant is instilled into each segmental bronchus upon diagnosis of IRI using fiber-optic bronchoscopy. This method significantly improves oxygenation, resolves infiltrates within 24 h, and offers a 100% survival rate at 19 months post-transplant in treated patients [18]. Established in lung transplant patients, bronchoscopic surfactant installation represents a cost-effective and relatively non-invasive alternative to ECMO, known to be invasive, labor-intensive, expensive, and frequently associated with significant refractory bleeding, renal failure, sepsis [18,52,53].

#### 3.4.5. ECLS Optimization and Invasive Device Management

Optimizing ECLS and pre-discharge invasive device management is pivotal in enhancing recovery and minimizing complications in cardiothoracic transplantation. Early postoperative extubation, particularly in lung transplant patients, has emerged as a critical component of ERAS protocols applicable to cardiothoracic transplant recipients. Studies suggest that extubation in the operating room within six hours post-transplant significantly improves outcomes by reducing ventilator-associated complications and expediting recovery [4,17]. Additionally, evidence demonstrates that early chest tube removal, ideally by the first postoperative day, enables faster respiratory and cardiomuscular rehabilitation, reducing the incidence of bronchopneumonia and other respiratory complications [20,21].

#### 3.4.6. Mobilization, Physiotherapy, and Psychotherapy

Prolonged bed rest is a well-documented risk factor for numerous adverse outcomes in postoperative patients, including physical deconditioning, reduced muscle mass, increased pulmonary complications such as atelectasis and pneumonia, and a heightened risk of ileus and venous thromboembolism [21,25]. To mitigate these risks and accelerate recovery, early mobilization, physiotherapy, and psychotherapy are integral components ERAS protocols for cardiac and lung transplantation.

These interventions typically commence on the day of surgery and include activities such as incentive spirometer use, deep breathing exercises, and coughing to promote pulmonary function and prevent complications. Passive range of motion exercises for the upper and lower extremities, as well as sitting upright, are standard practices aimed at reducing postoperative ileus and thromboembolic events [10,20]. Evidence supports initiating mobilization within 24 h post-surgery, with patients encouraged to sit at the edge of the bed within four hours of extubation and progress to sitting in a chair for 20 min, three times daily, starting on postoperative day one. When feasible, patients may also be encouraged to walk 100 steps in the ICU twice daily [21]. Early mobilization and physiotherapy reduce hospital stays, lower healthcare costs, and expedite the return to normal activities, all of which are critical to enhancing overall patient outcomes [10]. Eventually, long term exercise and mobilization is critical for a transplant patient. As heart transplantation leads to total denervation of the heart (both sympathetic and parasympathetic), with cardiac reinnervation occurring in a small subgroup of heart transplant recipients. Prevention and rehabilitations with physical and high intensity training is highly effective in heart recipients and should be tailored for each individual patient [54]. Furthermore, integrating psychosocial programs into the postoperative care plan is equally important. These programs focus on improving mental health, fostering social engagement, and promoting medication adherence, all of which are essential for long-term success in transplant recipients [12].

## 4. Discussion

The integration of ERAS protocols into cardiothoracic transplantation represents a transformative approach to improving outcomes in this high-risk patient population. Although initially developed for general surgery, ERAS principles—emphasizing multidisciplinary collaboration, evidence-based practices, and patient-centered care—have demonstrated significant potential when adapted to the complex challenges of heart and lung transplantation.

This systematic review highlights the efficacy of tailored interventions across the perioperative spectrum. Preoperative optimization, including nutritional supplementation, psychosocial support, and prehabilitation, lays a foundation for resilience against surgical stress. Intraoperative strategies such as minimally invasive techniques, advanced anesthetic management, and precise hemodynamic control enhance procedural outcomes while reducing complications. Postoperative protocols focused on opioid-sparing pain management, early mobilization, and nutritional recovery promote faster and more sustainable recoveries. Together, these measures aim to standardize care delivery, mitigate risks, and improve long-term survival.

However, the unique demands of cardiothoracic transplantation, including immunosuppression, organ rejection, and graft dysfunction, necessitate further research and innovation. While promising evidence supports the application of ERAS in lung transplantation, gaps remain in its implementation for heart transplantation. One such limitation is this gap in literature, resulting in this study relying on a significant number of review articles and cardiothoracic non-transplant techniques for our ERAS suggestions. With varying heterogenicity in studies such as review articles, case-control, cohort, and hypothetical cases, another limitation in this study design is the ability to directly compare outcome across studies. However, we also hope this also aids in adding depth to our proposed suggestions. Additionally, sources of bias may have been introduced throughout authorship screening, even with the help of a second and third reviewer.

Future studies should prioritize the validation of ERAS protocols, develop transplantation-specific adaptations, and explore novel technologies to address existing limitations. Additionally, due to the lack of literature on cardiothoracic transplant ERAS implementation, we recommend future studies to prioritize multicenter trials and outcome measures of transplant-specific ERAS.

A comprehensive, multidisciplinary approach is imperative to successfully implement ERAS protocols in cardiothoracic transplantation. By fostering collaboration among surgeons, anesthesiologists, intensivists, and allied health professionals, we can advance the paradigm of perioperative care. In doing so, ERAS has the potential to not only enhance clinical outcomes but also redefine recovery expectations for patients undergoing heart and lung transplantation.

## 5. Conclusions

ERAS protocols represent a promising and evolving framework for improving perioperative care and outcomes in cardiothoracic transplantation, a field marked by high morbidity, prolonged recovery, and complex patient needs. This study demonstrates that ERAS strategies have the potential to reduce complications, shorten hospital stays, and enhance functional and long-term outcomes. Key elements such as multidisciplinary prehabilitation, opioid-sparing analgesia, early mobilization, and targeted nutritional support emerge as consistent contributors to improved recovery. However, evidence specific to heart transplantation remains limited, necessitating extrapolation from broader cardiothoracic and lung transplant literature and underscoring a critical gap in current research. While this study provides important guidance for current practice, future efforts should focus on developing transplant-specific ERAS pathways. With thoughtful adaptation and multidisciplinary collaboration, ERAS has the potential to redefine perioperative care and recovery expectations for patients undergoing cardiothoracic transplantation.

## Figures and Tables

**Table 1:** Characteristics of final studies included. Cardiac, lung, refer to cardiac and lung surgery without transplantation methods. Cardiac transplant, lung transplant, refer to cardiac and lung transplantation surgery. Each article was characterized as presenting ERAS strategies for pre, intra and/or postoperative techniques. Author and design of each article was also documented.

Author, Year of Publication	Organ Focus: Cardiac, Lung, Cardiac Transplant (CT), Lung Transplant (LT)	Pre/Intra/Post-Operative ERAS Strategies	Design of Study
Wischmeyer, 2018 [9]	CT	Pre and post	Review
Wishahi, 2024 [10]	Cardiac, Lung	Pre, intra, and post	Review
Ander, 2023 [11]	CT, LT	Pre, intra, and post	Review
Martin, 2023 [12]	LT	Intra	Hypothetical Case
Shen, 2020 [13]	Cardiac, Lung	Pre, intra, and post	Review
Makaryus, 2018 [14]	Cardiac	Pre, intra, and post	Review
Lewis, 2022 [15]	LT	Intra	Case-control
Wu, 2024 [16]	LT	Intra	Retrospective Cohort
Habib, 2024 [17]	LT	Post	Retrospective Cohort
Bhatt, 2025 [4]	CT	Pre, post	Review
Kermeen, 2007 [18]	CT, LT	Post	Retrospective Cohort
Lobo, 2024 [19]	CT, LT	Post	Retrospective Cohort
Schneider, 2024 [20]	Cardiac	Pre, intra, and post	Case-control
Hendy, 2023 [21]	Cardiac	Pre, intra, and post	Retrospective Cohort
Ikeda, 2022 [22]	LT	Post	Randomized Controlled
Ahmed, 2022 [23]	LT	Intra	Retrospective Observational
Lewis, 2020 [24]	LT	Pre, intra, and post	Retrospective Cohort
Batchelor, 2019 [25]	Lung	Pre, intra, and post	Review
Williams, 2019 [26]	Cardiac	Pre, intra, and post	Case-control
Engelman, 2019 [7]	Cardiac	Pre, intra, and post	Review

**Figure A1:**
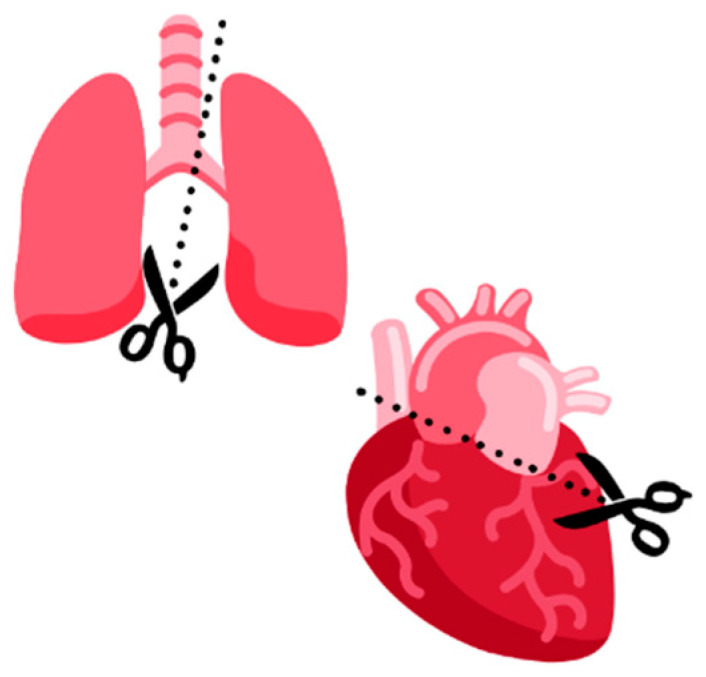
Central Picture. Cardiothoracic lung transplantation recovery optimized by enhanced recovery after surgery.

## Data Availability

All data sources have been cited in the references and were provided in Table 1.

## Supplementary Materials

The following supporting information can be downloaded at https://www.mdpi.com/article/10.3390/jcm15031179/s1, Supplementary Materials S1: PRISMA 2020 checklist [8].

## Abbreviations

The following abbreviations are used in this manuscript:

ECLS	Extracorporeal life support
ECMO	Extracorporeal Membrane Oxygenation
ERAS	Enhanced Recovery After Surgery
PGD	Primary graft dysfunction
IRI	Ischemic reperfusion injury
MILT	Minimally invasive lung transplantation

## References

[1] Bos S., Vos R., Van Raemdonck D.E., Verleden G.M. (2020). Survival in adult lung transplantation: Where are we in 2020?. Curr. Opin. Organ Transplant..

[2] Wilhelm M.J. (2015). Long-term outcome following heart transplantation: Current perspective. J. Thorac. Dis..

[3] Brown J.K., Singh K., Dumitru R., Chan E., Kim M.P. (2018). The Benefits of Enhanced Recovery After Surgery Programs and Their Application in Cardiothoracic Surgery. Methodist DeBakey Cardiovasc. J..

[4] Bhatt H.V., Fritz A.V., Feinman J.W., Subramani S., Malhotra A.K., Townsley M.M., Weiner M.M., Sharma A., Teixeira M.T., Nguyen B. (2025). The Year in Cardiothoracic and Vascular Anesthesia: Selected Highlights from 2024. J. Cardiothorac. Vasc. Anesth..

[5] Ljungqvist O., Scott M., Fearon K.C. (2017). Enhanced Recovery After Surgery: A Review. JAMA Surg..

[6] Joliat G.R., Ljungqvist O., Wasylak T., Peters O., Demartines N. (2018). Beyond surgery: Clinical and economic impact of Enhanced Recovery After Surgery programs. BMC Health Serv. Res..

[7] Engelman D.T., Ben Ali W., Williams J.B., Perrault L.P., Reddy V.S., Arora R.C., Roselli E.E., Khoynezhad A., Gerdisch M., Levy J.H. (2019). Guidelines for Perioperative Care in Cardiac Surgery: Enhanced Recovery After Surgery Society Recommendations. JAMA Surg..

[8] Page M.J., McKenzie J.E., Bossuyt P.M., Boutron I., Hoffmann T.C., Mulrow C.D., Shamseer L., Tetzlaff J.M., Akl E.A., Brennan S.E. (2021). The PRISMA 2020 statement: An updated guideline for reporting systematic reviews. BMJ..

[9] Wischmeyer P.E., Carli F., Evans D.C., Guilbert S., Kozar R., Pryor A., Thiele R.H., Everett S., Grocott M., Gan T.J. (2018). American Society for Enhanced Recovery and Perioperative Quality Initiative Joint Consensus Statement on Nutrition Screening and Therapy Within a Surgical Enhanced Recovery Pathway. Anesth. Analg..

[10] Wishahi M., Kamal N.M., Hedaya M.S. (2024). Enhanced recovery after surgery: Progress in adapted pathways for implementation in standard and emerging surgical settings. World J. Clin. Cases.

[11] Ander M., Mugve N., Crouch C., Kassel C., Fukazawa K., Izaak R., Deshpande R., McLendon C., Huang J. (2023). Regional anesthesia for transplantation surgery—A white paper part 1: Thoracic transplantation surgery. Clin. Transplant..

[12] Martin A.K., Reed A.K., Hoetzenecker K., Fessler J. (2023). How We Would Treat Our Own Lung Transplantation: A Multidisciplinary and International Perspective. J. Cardiothorac. Vasc. Anesth..

[13] Shen L., Tam C.W., Jones M.-M., Hoyler M., Ivascu N.S. (2020). Noteworthy Literature From 2019 for Cardiothoracic Critical Care. Semin. Cardiothorac. Vasc. Anesth..

[14] Makaryus R., Miller T., Gan T. (2018). Current concepts of fluid management in enhanced recovery pathways. Br. J. Anaesth..

[15] Lewis T.C., Sureau K., Katz A., Fargnoli A., Lesko M., Rudym D., Angel L.F., Chang S.H., Kon Z.N. (2022). Multimodal opioid-sparing pain management after lung transplantation and the impact of liposomal bupivacaine intercostal nerve block. Clin. Transplant..

[16] Wu R., Robayo V., Nguyen D.T., Chan E.Y., Chihara R., Huang H.J., Graviss E.A., Kim M.P. (2024). Enhanced recovery after surgery may mitigate the risks associated with robotic-assisted fundoplication in lung transplant patients. Surg. Endosc..

[17] Habib A., Gouchoe D.A., Rosenheck J.P., Mokadam N.A., Henn M.C., Nunley D.R., Ramsammy V., Whitson B.A., Ganapathi A.M. (2024). Early Extubation: Who Qualifies Postoperatively in Lung Transplantation?. J. Surg. Res..

[18] Kermeen F., McNeil K., Fraser J., McCarthy J., Ziegenfuss M.D., Mullany D., Dunning J., Hopkins P. (2007). Resolution of Severe Ischemia–Reperfusion Injury Post–Lung Transplantation After Administration of Endobronchial Surfactant. J. Heart Lung Transplant..

[19] Lobo C., Tufegdzic B. (2024). Postoperative pain management after thoracic transplantations. Curr. Opin. Anaesthesiol..

[20] Schneider C., Marguerite S., Ramlugun D., Saadé S., Maechel A.L., Oulehri W., Collange O., Mertes P.M., Mazzucotelli J.P., Kindo M. (2024). Enhanced Recovery After Surgery Program for Patients Undergoing Isolated Elective Coronary Artery Bypass Surgery Improves Postoperative Outcomes. J. Thorac. Cardiovasc. Surg..

[21] Hendy A., DiQuinzo C., O’Reilly M., Hendy A., Vician M., Theriault C., Chedrawy E., Hirsch G., Aliter H. (2023). Implementation of enhanced recovery in cardiac surgery: An experimental study with the control group. Asian Cardiovasc. Thorac. Ann..

[22] Ikeda M., Nakajima D., Oshima A., Oshima Y., Kayawake H., Tanaka S., Yamada Y., Yutaka Y., Ohsumi A., Hamaji M. (2022). The Effects of Early Postoperative Nutrition Support on Enhanced Recovery After Lung Transplantation. J. Heart Lung Transplant..

[23] Ahmed H., Zeschky C., Alayyar M., Husain M., Jothidasan A., Padukone A., Bello S., Marczin N., Smail H., Stock U. (2022). Long Term Outcomes of Minimally Invasive Lung Transplantation Compared to Clamshell Approach. J. Heart Lung Transplant..

[24] Lewis T.C., Sureau K., Katz A., Chen S., Angel L., Lesko M., Rudym D., Chang S., Kon Z. (2020). Enhanced Recovery and Opioid-Sparing Pain Management Following Lung Transplantation. J. Heart Lung Transplant..

[25] Batchelor T.J.P., Rasburn N.J., Abdelnour-Berchtold E., Brunelli A., Cerfolio R.J., Gonzalez M., Ljungqvist O., Petersen R.H., Popescu W.M., Slinger P.D. (2019). Guidelines for enhanced recovery after lung surgery: Recommendations of the Enhanced Recovery After Surgery (ERAS^®^) Society and the European Society of Thoracic Surgeons (ESTS). Eur. J. Cardiothorac. Surg..

[26] Williams J.B., McConnell G., Allender J.E., Woltz P., Kane K., Smith P.K., Engelman D.T., Bradford W.T. (2019). One-year results from the first US-based enhanced recovery after cardiac surgery (ERAS Cardiac) program. J. Thorac. Cardiovasc. Surg..

[27] Ljungqvist O., de Boer H.D., Balfour A., Fawcett W.J., Lobo D.N., Nelson G., Scott M.J., Wainwright T.W., Demartines N. (2021). Opportunities and Challenges for the Next Phase of Enhanced Recovery After Surgery: A Review. JAMA Surg..

[28] Michel-Cherqui M., Szekely B., Fessler J., Glorion M., Sage E., Le Guen M., Trichereau J., Vallée A., Fischler M. (2022). Feasibility and Usefulness of Self-Hypnosis in Patients Undergoing Double-Lung Transplantation During the Pre- and Postoperative Periods: A Randomized Study. J. Cardiothorac. Vasc. Anesth..

[29] Kyle U.G., Kossovsky M.P., Karsegard V.L., Pichard C. (2006). Comparison of tools for nutritional assessment and screening at hospital admission: A population study. Clin Nutr..

[30] Shander A., Corwin H.L., Meier J., Auerbach M., Bisbe E., Blitz J., Erhard J., Faraoni D., Farmer S.D., Frank S.M. (2023). Recommendations from the International Consensus Conference on Anemia Management in Surgical Patients (ICCAMS). Ann. Surg..

[31] Spahn D.R., Schoenrath F., Spahn G.H., Seifert B., Stein P., Theusinger O.M., Kaserer A., Hegemann I., Hofmann A., Maisano F. (2019). Effect of ultra-short-term treatment of patients with iron deficiency or anaemia undergoing cardiac surgery: A prospective randomised trial. Lancet.

[32] Wong J., Zoungas S., Wright C., Teede H. (2010). Evidence-based Guidelines for Perioperative Management of Diabetes in Cardiac and Vascular Surgery. World J. Surg..

[33] Tønnesen H., Nielsen P.R., Lauritzen J.B., Møller A.M. (2009). Smoking and alcohol intervention before surgery: Evidence for best practice. Br. J. Anaesth..

[34] Katsura M., Kuriyama A., Takeshima T., Fukuhara S., Furukawa T.A. (2015). Preoperative inspiratory muscle training for postoperative pulmonary complications in adults undergoing cardiac and major abdominal surgery. Cochrane Database Syst. Rev..

[35] Ahn H.J., Kim J.A., Lee A.R., Yang M., Jung H.J., Heo B. (2016). The Risk of Acute Kidney Injury from Fluid Restriction and Hydroxyethyl Starch in Thoracic Surgery. Anesth. Analg..

[36] Singh S.S.A., Das De S., Rushton S., Berry C., Al-Attar N. (2019). PREDICTA: A Model to Predict Primary Graft Dysfunction After Adult Heart Transplantation in the United Kingdom. J. Card. Fail..

[37] Hoetzenecker K., Benazzo A., Schwarz S., Keshavjee S., Cypel M. (2024). The Advent of Semi-Elective Lung Transplantation—Prolonged Static Cold Storage at 10 °C. Transpl. Int..

[38] Edwards F.H., Engelman R.M., Houck P., Shahian D.M., Bridges C.R., Society of Thoracic Surgeons (2006). The Society of Thoracic Surgeons Practice Guideline Series: Antibiotic Prophylaxis in Cardiac Surgery, Part I: Duration. Ann. Thorac. Surg..

[39] Thomas J., Barnes D., Chikwe J., Chen Q., Roach A., Peiris A., Rowe G., Gill G., Alhossan A., Berliner H. (2022). Impact of Minimally Invasive Lung Transplantation on Early Outcomes and Analgesia Use: A Matched Cohort Study. J. Heart Lung Transplant..

[40] Drees D., Tumin D., Miller R., Kirkby S., Bhalla T., Tobias J.D., Hayes D. (2018). Chronic opioid use and clinical outcomes in lung transplant recipients: A single-center cohort study. Clin. Respir. J..

[41] Thakuria L., Davey R., Romano R., Carby M.R., Kaul S., Griffiths M.J., Simon A.R., Reed A.K., Marczin N. (2016). Mechanical ventilation after lung transplantation. J. Crit. Care.

[42] Martin A.K., Harrison B.A., Fritz A.V., Landolfo K.P., Makey I.A., Sareyyupoglu B., Brown T.E., Johnson J.L., Pham S.M., Thomas M. (2020). Intraoperative management of a hybrid extracorporeal membrane oxygenation circuit for lung transplantation. J. Card. Surg..

[43] Levy J.H., Koster A., Quinones Q.J., Milling T.J., Key N.S. (2018). Antifibrinolytic Therapy and Perioperative Considerations. Anesthesiology.

[44] Simpson J.C., Bao X., Agarwala A. (2019). Pain Management in Enhanced Recovery after Surgery (ERAS) Protocols. Clin. Colon Rectal Surg..

[45] Barr L.F., Boss M.J., Mazzeffi M.A., Taylor B.S., Salenger R. (2020). Postoperative Multimodal Analgesia in Cardiac Surgery. Crit. Care Clin..

[46] Das Adhikary S., Prasad A., Soleimani B., Chin K.J. (2019). Continuous Erector Spinae Plane Block as an Effective Analgesic Option in Anticoagulated Patients After Left Ventricular Assist Device Implantation: A Case Series. J. Cardiothorac. Vasc. Anesth..

[47] Krishna S.N., Chauhan S., Bhoi D., Kaushal B., Hasija S., Sangdup T., Bisoi A.K. (2019). Bilateral Erector Spinae Plane Block for Acute Post-Surgical Pain in Adult Cardiac Surgical Patients: A Randomized Controlled Trial. J. Cardiothorac. Vasc. Anesth..

[48] Kushimoto S., Yamanouchi S., Endo T., Sato T., Nomura R., Fujita M., Kudo D., Omura T., Miyagawa N., Sato T. (2014). Body temperature abnormalities in non-neurological critically ill patients: A review of the literature. J. Intensive Care.

[49] Weimann A., Braga M., Harsanyi L., Laviano A., Ljungqvist O., Soeters P., Jauch K., Kemen M., Hiesmayr J., Horbach T. (2006). ESPEN Guidelines on Enteral Nutrition: Surgery including Organ Transplantation. Clin. Nutr..

[50] Martínez-Ortega A.J., Piñar-Gutiérrez A., Serrano-Aguayo P., González-Navarro I., Remón-Ruíz P.J., Pereira-Cunill J.L., García-Luna P.P. (2022). Perioperative Nutritional Support: A Review of Current Literature. Nutrients.

[51] Noble E.J., Harris R., Hosie K.B., Thomas S., Lewis S.J. (2009). Gum chewing reduces postoperative ileus? A systematic review and meta-analysis. Int. J. Surg..

[52] Glassman L.R., Keenan R.J., Fabrizio M., Sonett J.R., Bierman M.I., Pham S.M., Griffith B.P. (1995). Extracorporeal membrane oxygenation as an adjunct treatment for primary graft failure in adult lung transplant recipients. J. Thorac. Cardiovasc. Surg..

[53] Zenati M., Pham S.M., Keenan R.J., Griffith B.P. (1996). Extracorporeal membrane oxygenation for lung transplant recipients with primary severe donor lung dysfunction. Transpl. Int..

[54] Simonenko M., Hansen D., Niebauer J., Volterrani M., Adamopoulos S., Amarelli C., Ambrosetti M., Anker S.D., Bayes-Genis A., Ben Gal T. (2024). Prevention and rehabilitation after heart transplantation: A clinical consensus statement of the European Association of Preventive Cardiology, Heart Failure Association of the ESC, and the European Cardio Thoracic Transplant Association, a section of ESOT. Eur. J. Prev. Cardiol..

